# An Ionic 1,4-Bis(styryl)benzene-Based Fluorescent Probe for Mercury(II) Detection in Water via Deprotection of the Thioacetal Group

**DOI:** 10.3390/s16122082

**Published:** 2016-12-07

**Authors:** Van Sang Le, Ji-Eun Jeong, Huy Tuan Huynh, Jiae Lee, Han Young Woo

**Affiliations:** 1 Department of Cogno-Mechatronics Engineering, Pusan National University, Busan 46241, Korea; vansang@pusan.ac.kr (V.S.L.); huynhhuytuan510@gmail.com (H.T.H.); 2 Department of Chemistry, Korea University, Seoul 02841, Korea; jieunj@korea.ac.kr (J.-E.J.); brant0212@korea.ac.kr (J.L.)

**Keywords:** mercury, conjugated oligoelectrolyte, aqueous media, chemosensor, fluorescent sensor

## Abstract

Highly sensitive and selective mercury detection in aqueous media is urgently needed because mercury poisoning usually results from exposure to water-soluble forms of mercury by inhalation and/or ingesting. An ionic conjugated oligoelectrolye (M1Q) based on 1,4-bis(styryl)benzene was synthesized as a fluorescent mercury(II) probe. The thioacetal moiety and quaternized ammonium group were incorporated for Hg^2+^ recognition and water solubility. A neutral Hg^2+^ probe (M1) was also prepared based on the same molecular backbone, and their sensor characteristics were investigated in a mixture of acetonitrile/water and in water. In the presence of Hg^2+^, the thioacetal group was converted to aldehyde functionality, and the resulting photoluminescence intensity decreased. In water, M1Q successfully demonstrated highly sensitive detection, showing a binding toward Hg^2+^ that was ~15 times stronger and a signal on/off ratio twice as high, compared to M1 in acetonitrile/water. The thioacetal deprotection by Hg^2+^ ions was substantially facilitated in water without an organic cosolvent. The limit of detection was measured to be 7 nM with a detection range of 10–180 nM in 100% aqueous medium.

## 1. Introduction

Conjugated oligoelectrolytes (COEs) are characterized by a π-conjugated main backbone with ionic side-chains (cationic or anionic), having unique electrical and optical properties due to effective π conjugation as well as good solubility in highly polar media such as water. In these COE structures, the bandgap is in the range of UV-Vis wavelengths because π and π* orbitals constitute the highest occupied molecular orbital (HOMO) and the lowest unoccupied molecular orbital (LUMO), enabling their applications in colorimetric and/or fluorescent sensors and imaging [1]. Based on the useful electrical and optical characteristics of COEs, various types of chemo- and bioassays have been successfully demonstrated using COEs as a signaling platform for a wide range of target materials such as metal ions, deoxyribonucleic acid (DNA), ribonucleic acid (RNA), peptides, and antibodies [2,3,4,5].

Mercury is a very toxic and hazardous material by accumulating through the food chain, seriously influencing human health with fatal damages in several human organs, such as the brain, the heart, and the kidney, via conversion into methylmercury [6,7,8,9]. We can be easily exposed to mercury in neighboring environments including coal and gold mining, fossil fuel combustion, chemical manufacturing, volcanic emission, and forest fires [10,11,12]. Due to its obvious hazard, mercury is strictly banned in electrical and electronic equipment by the European Union′s Restriction on Hazardous Substances (RoHS) [13]. The mercury ion concentration in drinking water is also strictly regulated by the World Health Organization (WHO) [14] and the Environmental Protection Agency (EPA) [15]. Various techniques, including atomic absorption spectroscopy, cold vapor atomic fluorescence spectrometry, and gas chromatography, have been developed for sensitive and selective detection of metal ions; however, these methods require complicated sample preparation and sophisticated instrumentation. Fluorescence-based detection is one of the most widely used methods due to its simplicity of measurement, rapid response, and high sensitivity [16,17,18,19,20,21]. Many researchers have studied conjugated structure-based fluorescent probe molecules (ferrocene, rhodamine, naphthalimide, boron-dipyrromethene (BODIPY), anthracene, and porphyrin-based molecules) for mercury detection with high sensitivity and selectivity [22,23,24,25].

Mercaptans (or thiols) are well-known mercury-capturing substances due to their strong binding affinity toward mercury ions [26]. Based on the mercury(II)-promoted thioacetal deprotection, several Hg^2+^ detection assays have been reported [27,28,29,30,31,32,33]. Zhen Li’s group developed an ethylthio-posessing, azobenzene- or triphenylamine-based chemosensor for Hg^2+^ detection, utilizing intramolecular charge transfer (ICT) as a sensory mechanism [30,31]. In the presence of Hg^2+^, electron donating alkylthioacetal groups were converted to an electron-withdrawing aldehyde group, resulting in changes in ICT interaction through the molecule. By differentiating the electron donating groups, the sensitivity of the sensor system was successfully modulated, showing a 10–400 nM limit of detection (LOD). However, most previous Hg^2+^ assays demonstrated Hg^2+^ detection in organic solvents such as tetrahydrofuran (THF), MeOH, and mixed organic/water due to the poor water solubility of fluorescent probes [29,30,31,32,33]. Highly sensitive and selective Hg^2+^ detection in aqueous media is urgently needed because mercury poisoning usually results from the exposure to water-soluble forms of mercury by inhalation and/or ingesting.

In this contribution, a water-soluble fluorescent conjugated oligoelectrolyte, M1Q (as an aqueous Hg^2+^ probe) based on 1,4-bis(styryl)benzene was synthesized, and the Hg^2+^ detection characteristics were investigated in a 100% aqueous solution without the aid of organic solvents. The neutral precursor probe (M1) was also studied as a Hg^2+^ probe in a mixture of acetonitrile (CH_3_CN) and water, and the detailed sensor characteristics were compared in a mixture of CH_3_CN/water and 100% aqueous media. The ethylthioacetal group as a mercury sensitive site and ionic side-chains containing quaternized ammonium bromide (for water solubility) were incorporated at both termini of the π-conjugated backbone. In the presence of Hg^2+^ ions, chemical transformation of thioacetal into aldehyde occurs; photoluminescence (PL) spectral changes were measured with changing [Hg^2+^]. As compared to the detection in CH_3_CN/water, the aqueous detection system (M1Q) showed a binding toward Hg^2+^ that was approximately 15 times stronger, resulting in a stiff response curve with a smaller dissociation constant (*K*_d_) of 32 nM (vs. 570 nM for M1) and a signal on/off ratio that was approximately twice as high.

## 2. Materials and Methods

### 2.1. General

All chemical reagents were purchased from Aldrich Co. (Seoul, Korea) and used without further purification. ^1^H- and ^13^C-NMR spectra were recorded by JEOL FT NMR system (JNM-AL300, JEOL, Peabody, MA, USA) operating at 300 MHz and 75 MHz, respectively. UV-Vis spectra were measured with a Jasco V-630 UV-Vis spectrophotometer (JASCO International Co., LTD., Tokyo, Japan) and the PL spectra were obtained on a Jasco FP-6500/FP-8600 spectrofluorometer (JASCO International Co., LTD., Tokyo, Japan). The PL quantum yield of probe molecules was measured relative to fluorescein in water at pH = 10 as a standard [34,35].

### 2.2. Mercury(II) Ion Detection Protocol

Stock solutions (1 × 10^−3^ M) of M1 in acetonitrile and M1Q in deionized water was prepared. An amount of 2 μL of the stock solution of M1 was diluted into 2 mL of CH_3_CN/water (8:2), and 2 μL of the stock solution of M1Q was diluted into 2 mL of water to make [M1] = [M1Q] = 1.0 × 10^−6^ M. The PL spectra were measured with increasing [Hg^2+^]. After shaking the solution for 5 min, PL spectra were measured by excitation at 390 nm. The same procedures were repeated in the presence of KCl, NaCl, LiCl, AgNO_3_, Hg(NO_3_)_2_, CaCl_2_, CuCl_2_, MgCl_2_, FeCl_2_, AlCl_3_, PbCl_2_, and CdCl_2_ to assess the selectivity against other metal ions.

### 2.3. Synthesis

Compounds **2** and **4** were synthesized by following previous reports [36,37].

#### 2.3.1. 4-Bis(ethylthio)Methylbenzaldehyde (**5**)

A 250 mL round bottom flask was prepared and dried under vacuum. A mixture of terephthalaldehyde (5.00 g, 37.2 mmol) and ethanethiol (6.07 mL, 82.1 mmol) was dissolved in 60 mL of dichloromethane (DCM). The solution was stirred for 30 min under N_2_ at 0 °C. Then, the boron trifluoride etherate (BF_3_O(C_3_H_5_)_2_) solution (10.0 mL, 82.1 mmol) was added to initiate the reaction, and the mixture was stirred at room temperature for 24 h. After the reaction was completed, the mixture was neutralized by adding excess NaHCO_3_ to adjust the pH around 7.0–8.0. The product was extracted with DCM, dried over anhydrous magnesium sulfate, and purified by silica gel column chromatography using hexane/DCM (5:4, by volume) as an eluent. The final product was dried under vacuum (3 g, yield: 33%). ^1^H-NMR (CDCl_3_, 300 MHz): *δ* (ppm) 1.08–1.17 (t, 6H), 2.40–2.60 (m, 4H), 4.90 (s, 1H), 7.49–7.61 (d, 2H), 7.73–7.84 (d, 2H), 9.99 (s, 1H). ^13^C-NMR (CDCl_3_, 75 MHz): *δ* (ppm) 14.1, 25.1, 26.1, 35.5, 51.9, 128.2, 129.3, 129.9, 135.6, 147.4, 191.6, 191.8.

#### 2.3.2. Synthesis of Neutral 1,4-Bis(Styryl)Benzene-Based Mercury Probe (**M1**)

Into a 100 mL two neck round bottom flask, 4-*N*,*N*-bis(6′-bromohexyl)aminobenzaldehyde (0.279 g, 0.623 mmol), 1,4-bis[(diethylphosphoryl)methyl]benzene (0.236 g, 0.623 mmol), compound 5 (0.150 g, 0.623 mmol), and 20 mL anhydrous THF were added, and the reaction mixture was stirred at 0 °C. Then, potassium *t*-butoxide (0.35 g, 3.12 mmol) in 20 mL of anhydrous THF was slowly injected. The solution was stirred at 0 °C for 1 h. The product was extracted with DCM, dried over anhydrous magnesium sulfate, and purified by silica gel column chromatography using hexane/DCM (5:2 by volume) as eluent. After drying under vacuum, 140 mg of a neutral precursor was obtained (yield: 30%). ^1^H-NMR (CDCl_3_, 300 MHz): *δ* (ppm) 1.20–1.25 (m, 6H), 1.31–1.38 (m, 4H), 1.42–1.52 (m, 4H), 1.54–1.61 (m, 4H), 1.73–1.82 (m, 4H), 2.49–2.64 (m, 4H), 3.24–3.29 (m, 4H), 3.51–3.55 (t, 4H), 4.93 (s, 1H), 6.59–6.62 (d, 2H), 6.84–7.07 (dd, 2H), 7.08 (s, 2H), 7.36–7.48 (m, 10H). ^13^C-NMR (CDCl_3_, 75 MHz): *δ* (ppm) 135.51, 128.75, 128.06, 127.84, 127.29, 126.80, 126.63, 126.54, 126.22, 111.78, 52.21, 52.07, 50.92, 44.96, 32.54, 27.20, 26.75, 26.24, 14.30.

#### 2.3.3. Synthesis of Quaternized Ionic 1,4-Bis(Styryl)Benzene-Based Mercury (II) Probe (**M1Q**)

M1 (120 mg, 0.16 mmol) was dissolved in 10 mL of THF, and 5.0 mL of 30% aqueous trimethylamine solution was added. The mixture was stirred at room temperature for 24 h. A small amount of methanol was added to the above solution to re-dissolve the precipitate. An additional 5.0 mL of 30% aqueous trimethylamine solution was added again, and the resulting solution was stirred for another 24 h at room temperature. After the reaction was completed, excess trimethylamine and the solvent were distillated out under reduced pressure. The crude product was dissolved in a small amount of methanol and precipitated into cold diethyl ether. The precipitate was collected, washed with diethyl ether, and dried under vacuum to produce cationic fluorophore (yield: 90%). ^1^H-NMR (300 MHz, DMSO-d_6_): *δ* (ppm) 1.11–1.15 (t, 6H), 1.31 (m, 8H), 1.52 (m, 4H), 1.65 (m, 4H), 2.49–2.53 (m, 4H), 3.03 (s, 18H), 3.27 (m, 8H), 5.14 (s, 1H), 6.61 (m, 2H), 6.88–7.14 (dd, 2H), 7.09–7.14 (s, 2H), 7.39–7.56 (m, 10H). ^13^C-NMR (CDCl_3_, 75 MHz): *δ* (ppm) 147.65, 140.51, 137.94, 137.11, 135.65, 129.30, 128.91, 128.39, 127.55, 127.35, 126.90, 126.57, 124.88, 124.38, 123.04, 122.25, 121.54, 111.95, 65.64, 54.85, 52.59, 51.55, 50.43, 27.16, 26.41, 26.17, 22.58, 14.87.

## 3. Results

**Scheme 1:**
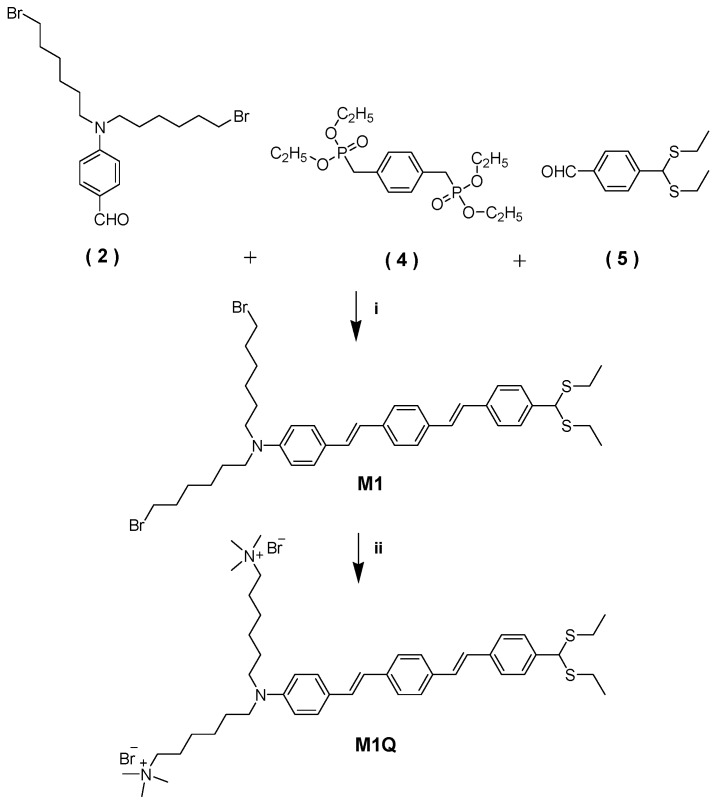
Synthetic scheme: (**i**) Potassium *t*-butoxide, THF, 0 °C, 1 h; (**ii**) 30% aq trimethylamine, THF/MeOH, room temperature, 48 h.

**Figure 1:**
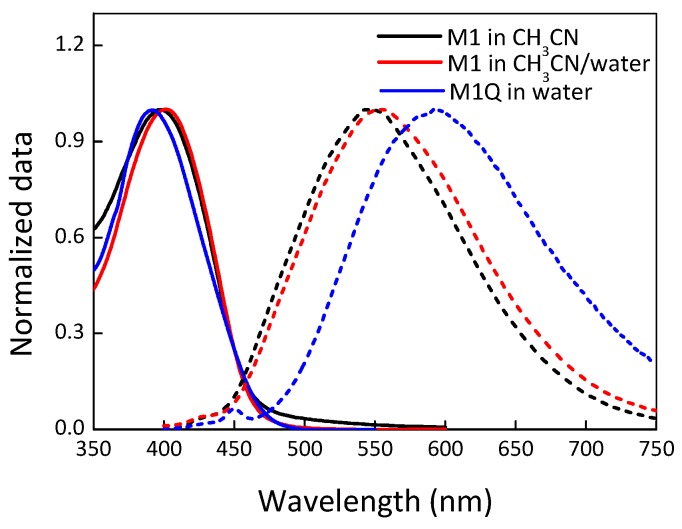
Normalized absorption (solid) and PL spectra (dash) of M1 and M1Q. Excitation at 390 nm. [M1] = [M1Q] =1.0 × 10^−6^ M.

The neutral mercury(II) probe (M1) was synthesized in ~30% yield via the Wittig-Honer-Emmons reaction of 4-*N*,*N*-bis(6′-bromohexyl)aminobenzaldehyde, 1,4-bis[(diethylphosphoryl)methyl]benzene, and 4-bis(ethylthio)methylbenzaldehyde (Scheme 1). The water-soluble cationic Hg^2+^ probe (M1Q) was prepared in a yield of ~90% by a simple quaternization reaction of M1 and trimethylamine in a mixture of methanol and THF [36,37]. The UV-Vis and PL spectra in CH_3_CN, water, and a solvent mixture of CH_3_CN/water are shown in Figure 1. The neutral probe, M1 shows the maximum absorption and emission at *λ*_abs_ = 397 nm and *λ*_PL_ = 545 nm (PL quantum yield (*Φ*_PL_) = 58.7%) in CH_3_CN, while the maximum wavelength is shifted to *λ*_abs_ = 402 nm and *λ*_PL_ = 554 nm (*Φ*_PL_ = 56.4%) in a mixture of CH_3_CN/water (8:2 by volume). M1Q shows the maximum absorption and PL at *λ*_abs_ = 392 nm and *λ*_PL_ = 592 nm (*Φ*_PL_ = 11.6%) in water. The PL spectra display a clear solvatochromism, showing a gradual red-shift with increasing solvent polarity. Molar absorption coefficients (*ε*_max_) were determined to be 5.35 × 10^4^ M^−1^ cm^−1^ in CH_3_CN, 5.52 × 10^4^ M^−1^·cm^−1^ in CH_3_CN/water for M1, and 3.85 × 10^4^ M^−1^·cm^−1^ in water for M1Q at each maximum absorption wavelength (Table 1). The ionic M1Q shows a largely increased Stokes shift of ~200 nm in water and decreased PL quantum efficiency due to enhanced nonradiative relaxations in highly polar solvent of water [38,39,40,41,42,43].

**Scheme 2:**
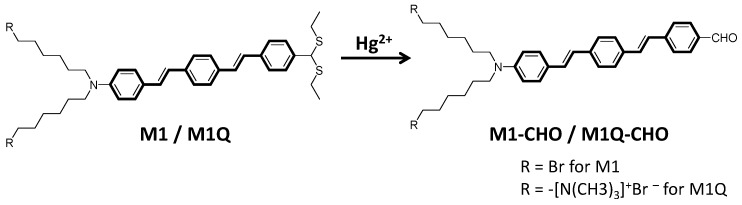
Structural transformation of thioacetal into aldehyde in the presence of Hg^2+^ ions.

Upon addition of Hg^2+^ ions, the terminal ethylthio group was reported to be converted to aldehyde to form M1-CHO and M1Q-CHO (Scheme 2) [30,32]. To confirm the formation of aldehyde in the presence of Hg^2+^, ^1^H-NMR spectra of M1 and M1Q were measured before and after the addition of Hg^2+^ ions. As shown in Figure S1, three proton (near sulfur atom) peaks were observed at *δ* = 1.20–1.25 ppm (6H, triplet –CH_3_), 2.49–2.64 ppm (4H, multiplet, –S-CH_2_–), and 4.93 ppm (1H, singlet, –CH=) in the ^1^H-NMR spectrum of M1Q. Upon the addition of Hg^2+^ ions, the above three peaks disappeared and a new proton peak appeared at *δ* = 9.97 ppm (1H, singlet), indicating that the thioacetal functional group in M1Q is transformed into aldehyde via the deprotection reaction (Figure S2). With regard to M1, the same chemical transformation was observed with the addition of Hg^2+^ ions (Figures S3 and S4), where a new proton peak from –CHO group was measured at *δ* = 9.97 ppm (1H, singlet).

The photophysical properties such as fluorescence quantum yield and ICT are strongly dependent on the electronic structure of molecules based on different push-pull abilities between donor and acceptor. Upon addition of Hg^2+^, the electron-donating ethylthio group at the end of both Hg^2+^ probe molecules (M1 and M1Q) is converted to the electron-withdrawing aldehyde group (M1-CHO and M1Q-CHO). Overall ICT interaction changes form M1 (or M1Q) with a donor-acceptor-donor (D-A-D) structure to M1-CHO (or M1Q-CHO) with a D-A structure before and after reacting with Hg^2+^. It has already been reported that the asymmetrical D-A structure, compared to that of symmetrical D-A-D structure, has a larger change in charge distribution upon excitation [44]. Via transformation of ethythio into aldehyde, the ICT interaction and the resulting electronic structures are expected to change significantly, inducing a spectral change in the PL emission with a strongly quenched signal. The PL intensity of M1Q in water decreased significantly (*Φ*_PL_ = 11.6% → 0.78%) with the addition of Hg^2+^ ions (2.0 × 10^−6^ M), and the maximum PL wavelength was blue-shifted from 592 nm to 584 nm (Table 1). M1 also showed a similar trend in CH_3_CN/water, showing suppressed PL efficiency (56.4% → 22.1%) with a slight change in *λ*_PL_ (from 554 nm to 560 nm). Interestingly, the clear improvement of signal on/off ratio with/without Hg^2+^ was measured to be ~10 in M1Q (water), compared to ~5 of M1 (CH_3_CN/water).

**Figure 2:**
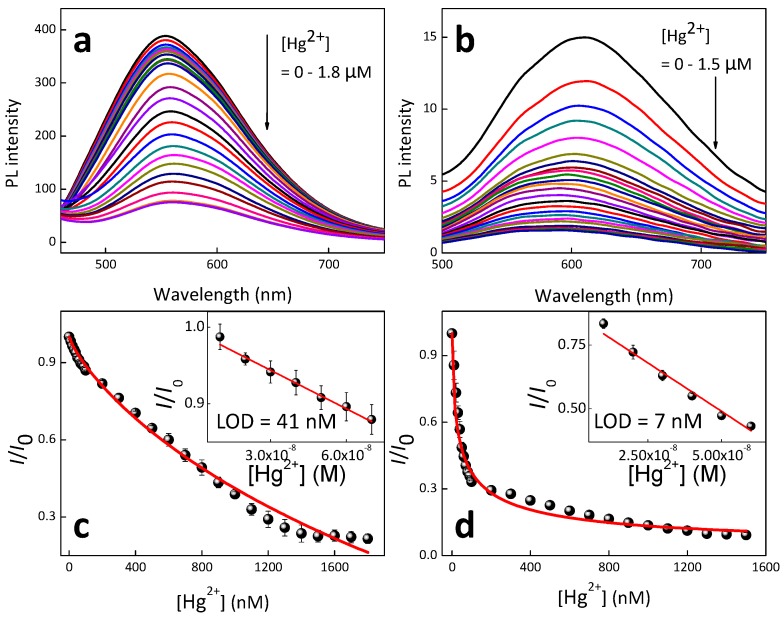
(**a**,**b**) PL spectra of M1 in CH_3_CN/water (8:2) and M1Q in water with increasing [Hg^2+^]. (**c**,**d**) Normalized PL intensity (*I*/*I*_0_) of M1 and M1Q with changing [Hg^2+^] (*I*/*I*_0_, where *I* and *I*_0_ are the PL intensity at *λ*_PL_ in the presence and absence of Hg^2+^ ions). Excitation at 390 nm. Inset: LOD determination. The error bars represent the standard deviation of six independent measurements. [M1] = [M1Q] = 1.0 × 10^−6^ M.

**Figure 3:**
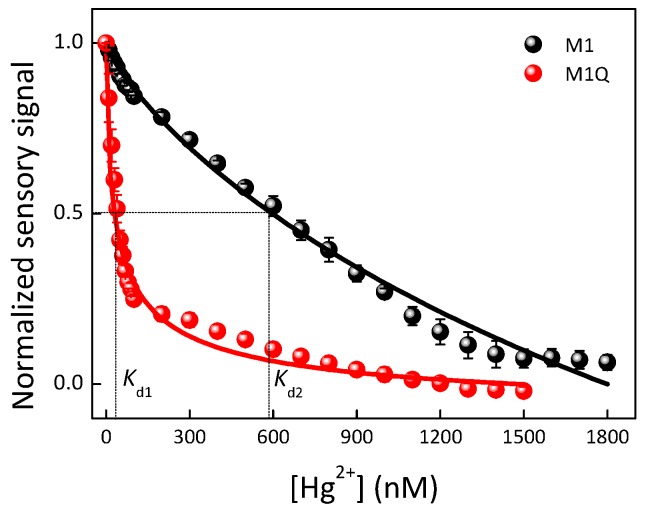
Titration curves and dissociation constants of M1 in CH_3_CN/water and M1Q in water.

To investigate the assay characteristics, the PL responses of M1 and M1Q versus [Hg^2+^] in CH_3_CN/water and in water (at pH = 7) were measured (Figure 2). The neutral probe (M1) needs the help of additional organic solvent (CH_3_CN) to be dissolved due to a lack of water solubility (CH_3_CN:water = 8:2 by volume). Each PL spectrum was obtained at [M1] = [M1Q] = 1.0 × 10^−6^ M with increasing [Hg^2+^] after 5 min of shaking. With increasing [Hg^2+^], the PL intensity of M1 and M1Q decreased gradually by forming M1-CHO or M1Q-CHO. The normalized PL intensity (*I*/*I*_0_, where *I* and *I*_0_ are the PL intensity at *λ*_PL_ in the presence and absence of Hg^2+^ ions) vs. [Hg^2+^] shows a hyperbolic transition, showing a detection range of 50–1360 nM for M1 and 10–180 nM for M1Q, respectively. The detection range was determined by fitting the titration curve using the Hill equation [45,46], where *I*/*I*_0_ transits from 10% to 90% of its signal output. The LOD was calculated by the following equation: LOD = 3.3 × *σ*/slope (*σ* is the standard deviation of six independent measurements of blank samples; slope was measured from the linearly fitted response curve (*I*/*I*_0_ vs. [Hg^2+^]). Insets of Figure 2c,d show the linear range of titration curves as a function of [Hg^2+^], showing LOD = 41 nM and 7 nM for M1 and M1Q, respectively. By looking into the both spectral curves of M1 and M1Q carefully, M1Q shows a much sharper change in the PL intensity as a function of [Hg^2+^], where M1 shows only an ~15% decrease in PL intensity with [Hg^2+^] = 0–100 nM, while an ~70% decrease in PL intensity was observed for M1Q. This indicates that M1Q in water can detect the smaller changes in [Hg^2+^] compared to M1 in CH_3_CN/water, which is closely related to the dissociation constant (*K*_d_) of M1 (or M1Q) and Hg^2+^. By measuring the Hg^2+^ concentration when the *I*/*I*_0_ ratio decreases to half of the original value without Hg^2+^, the *K*_d_ value was determined to be 570 nM (binding constant (*K*_a_) = 2 × 10^6^ M^−1^) for M1 and 32 nM (*K*_a_ = 3 × 10^7^ M^−1^) for M1Q, respectively (Figure 3). The binding affinity of M1Q toward Hg^2+^ was ~15 times higher than M1, indicating a stronger interaction between Hg^2+^ and ionic M1Q in water. Moreover, efficient binding between Hg^2+^ and M1Q results in an early saturation of sensory signals, showing a short detection range of Hg^2+^ ions, relative to M1 in CH_3_CN/water.

**Figure 4:**
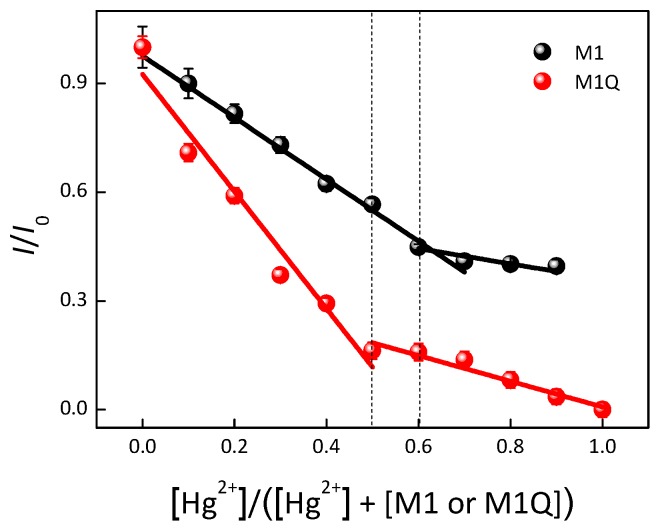
Job plots of M1 in CH_3_CN/water and M1Q in water.

The probe-Hg^2+^ binding stoichiometry was also studied by the Job plot experiment for M1 in CH_3_CN/water and M1Q in water (Figure 4). The Job plot is widely used in analytical chemistry to determine the stoichiometry of a binding event [47]. The PL signal was measured by changing [Hg^2+^]/([Hg^2+^] + [M1] or [M1Q]) for the M1 and M1Q sensory systems. Figure 4 shows a saturation point at [Hg^2+^]/([Hg^2+^] + [M1] or [M1Q]) = 0.6 and 0.5 for M1 and M1Q, indicating the 1:1.5 and 1:1 probes: the Hg^2+^ binding ratios for M1 in CH_3_CN/water and for M1Q in water.

The Hg^2+^ detection was also carried out in water by varying the pH of the solution (pH = 4, 7, 10). In an acidic condition (pH = 4), the spectral responses were different compared to those at pH = 7 and 10 because of the protonation of amine and ethylthio groups (Figure S5), which may have significantly influenced the binding event of M1Q and Hg^2+^, and the resulting thioacetal deprotection reaction at pH = 4. The Hg^2+^ detection characteristics of M1Q at pH = 10 were similar to those at pH = 7, showing a similar *K*_d_ of 31 nM, LOD of 2 nM, and 1:1 binding stoichiometry in the Job plot (Figure S6). However, a smaller signal on/off ratio (~5) was measured at pH = 10 compared to that (~10) at pH = 7, because of the higher PL intensity of M1Q-CHO (at the signal off state) under basic condition. To take advantages of the high signal on/off ratio, all the PL experiments in water were performed in deionized water at pH = 7.0 to maximize the sensory properties.

The sensor characteristics of M1Q in real samples (Han River and tap water) were also investigated. Before PL experiments, all real samples were first filtered with a 0.2 μm syringe filter. There were no detectable mercuric ions in the Han River or in tap water, and the PL characteristics of M1Q were investigated with the addition of Hg^2+^ (Figure S7). The normalized PL intensity versus [Hg^2+^] shows a sigmoidal transition curve, exhibiting *K*_d_ = 72 nM and 47 nM in the Han River and in tap water, respectively. The LOD was approximately determined to be 10 nM in the Han River and 2 nM in tap water, respectively (Figure S7c,d inset). Similar sensory characteristics were measured in real samples compared to those in deionized water (pH = 7). The sharp change in the PL intensity as a function of [Hg^2+^] (50%–60% of PL intensity decrease with [Hg^2+^] = 0–100 nM) was still observed, facilitating the successful detection in real samples.

**Figure 5:**
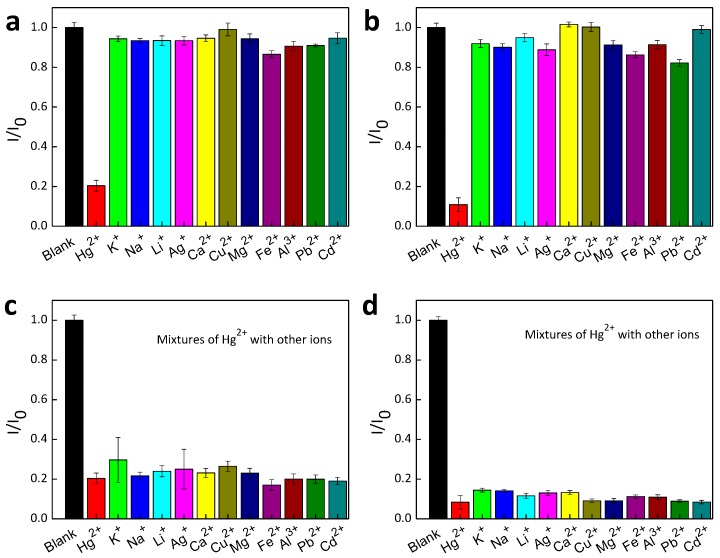
Selectivity test for M1 (**a**,**c**) and M1Q (**b**,**d**) in the presence of single (**a**,**b**) and co-existing (**c**,**d**) metal ions. The error bars represent the standard deviation of three independent measurements. [M1] = [M1Q] = 1.0 × 10^−6^ M, [metal ion] = 2.0 × 10^−6^ M.

Finally, the selectivity test of M1 and M1Q toward Hg^2+^ was performed in the presence of other metal ions (Figure 5). A negligible decrease in the PL signal of both M1 and M1Q was observed upon the addition of other metal ions (2.0 × 10^−6^ M), suggesting the high selectivity toward Hg^2+^ over other metal ions including K^+^, Na^+^, Li^+^, Ag^+^, Ca^2+^, Cu^2+^, Mg^2+^, Fe^2+^, Al^3+^, Pb^2+^, and Cd^2+^. The significant PL intensity drop with Hg^2+^ in the presence of coexisting metal ions implies a dominant binding between M1 (or M1Q) and Hg^2+^, with a negligible influence from other metal ions (Figure 5c,d). Figure S8 shows the selectivity data of M1Q against a range of metal ions in real samples. A decrease in the PL signal was observed only for Hg^2+^ in both the Han River and tap water because of a strong binding affinity between the ethylthio group and the Hg^2+^ ions. It is also noteworthy that the stronger binding between M1Q and Hg^2+^ provides a higher on/off ratio in sensory responses, although both M1 and M1Q demonstrate successfully high selectivity.

## 4. Conclusions

We designed and synthesized a cationic, quasi-linear conjugated fluorescent probe (M1Q) for mercury(II) detection in aqueous media. To detect the water-soluble form of mercury in water, ionic alkyl chains and the thioacetal group were incorporated at both termini of the conjugated backbone as water-solubilizing and mercury recognizing sites. A neutral mercury(II) probe (M1) was also prepared based on the same conjugated framework. In the presence of Hg^2+^, thioacetal was converted to aldehyde functionality, resulting in a slight shift in the PL spectrum with the decrease in PL intensity. Compared to M1, the thioacetal/mercury binding was substantially enhanced by ~15 times for M1Q in water, showing a smaller *K*_d_ of 32 nM compared to that (570 nM) for M1 in CH_3_CN/water. The detection range was narrower in water (10–180 nM) relative to that (50–1360 nM) in CH_3_CN/water. The thioacetal deprotection by Hg^2+^ ions was clearly facilitated in water without an organic cosolvent. The signal on/off ratio (*I*_on_/*I*_off_) was also approximately twice as high in water. M1Q showed good selectivity toward Hg^2+^ without any influence from other coexisting metal ions. This study emphasizes the molecular design of water-soluble fluorescent probes that can detect mercury(II) in aqueous solutions without the aid of an organic cosolvent.

## Figures and Tables

**Table 1:** Optical properties of M1, M1-CHO, M1Q, and M1Q-CHO.

Materials	Solvent	*λ*_abs_ (nm)	*λ*_PL_ (nm)	*Φ*_PL_ (%)	*ε*_max_ (M^−1^·cm^−1^)
M1	CH_3_CN	397	545	58.7	5.35 × 10^4^
	CH_3_CN/water	402	554	56.4	5.52 × 10^4^
M1-CHO	CH_3_CN/water	410	560	22.1	5.37 × 10^4^
M1Q	Water	392	592	11.6	3.85 × 10^4^
M1Q-CHO	Water	409	584	0.78	1.33 × 10^4^

## Supplementary Materials

The followings are available online at http://www.mdpi.com/1424-8220/16/12/2082/s1: Figures S1–S4: ^1^H-NMR spectra; Figure S5: PL spectra of M1Q at pH 4 and pH 10; Figure S6: Job plots of M1Q in water at pH 7 and pH 10; Figure S7: PL spectra of M1Q in real samples; Figure S8: Selectivity test of M1Q in real samples, Explanation of sensor properties.
