# Supplementary material for: An Ionic 1,4-Bis(styryl)benzene-Based Fluorescent Probe for Mercury(II) Detection in Water via Deprotection of the Thioacetal Group
Source: Sensors (Basel). 2016 Dec 7;16(12):2082. doi: 10.3390/s16122082 (PMC5191063; doi:10.3390/s16122082)
Supplement: Supplementary file 1 [file sensors-16-02082-s001.pdf]

Supplementary Materials: An Ionic 1,4-Bis(styryl)benzene-Based Fluorescent Probe for Mercury(II) Detection in Water via Deprotection of the Tthioacetal Group

Van Sang Le, Ji-Eun Jeong, Huy Tuan Huynh, Jiae Lee and Han Young Woo

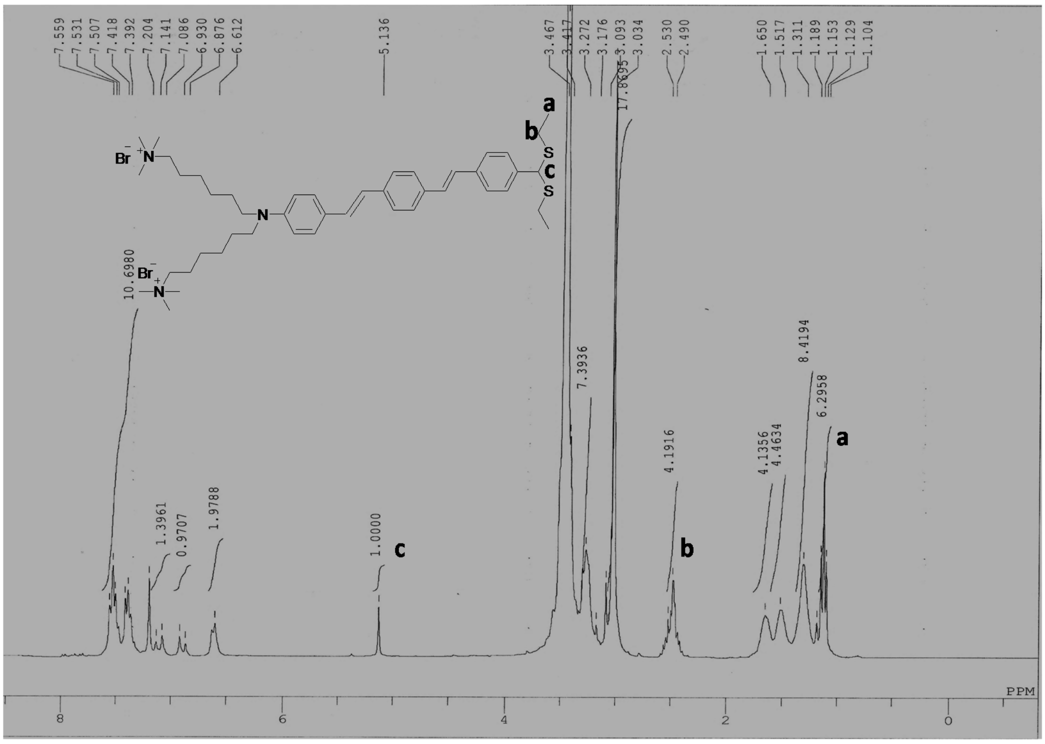

Figure S1. ^{1}H-NMR spectrum of M1Q in dimethyl sulfoxide-d$_6$.

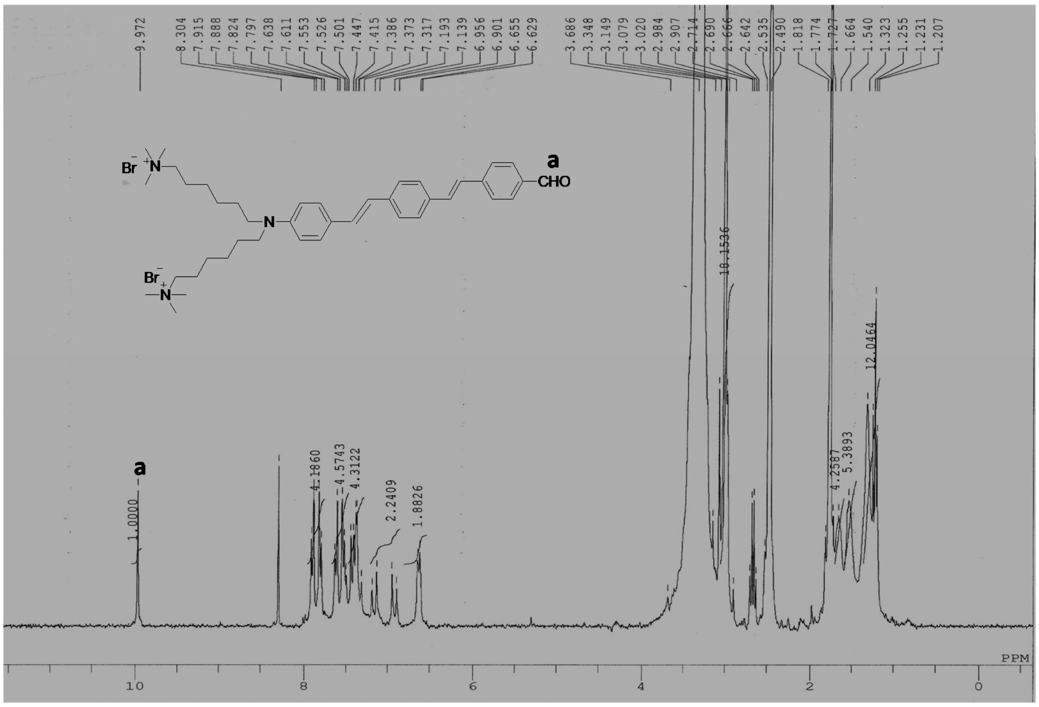

Figure S2. ^{1}H-NMR spectrum of M1Q-CHO in dimethyl sulfoxide-d$_6$.

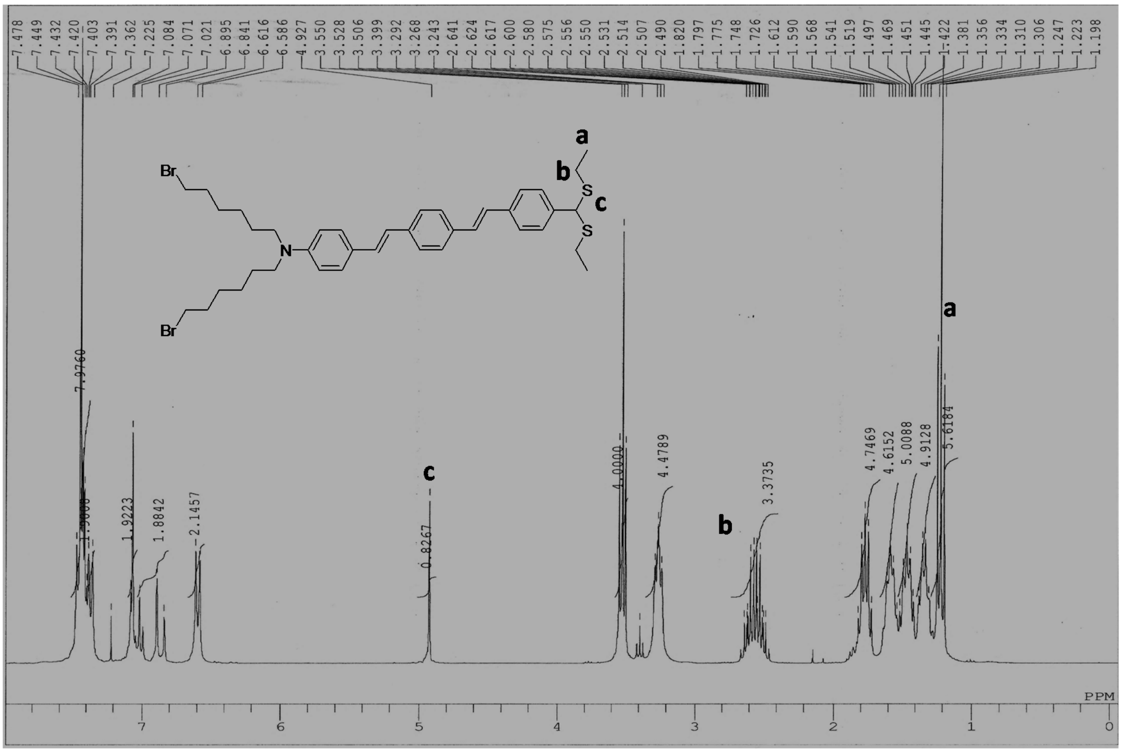

Figure S3. ^{1}H-NMR spectrum of M1 in $CDCl_3$.

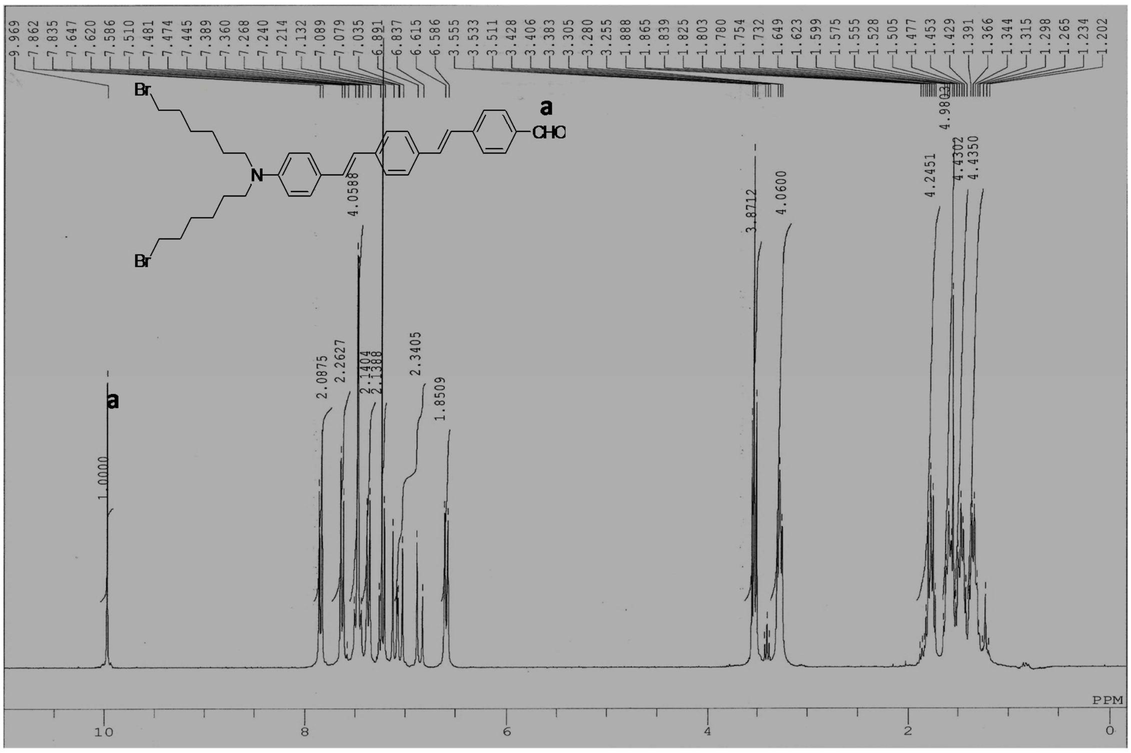

Figure S4. ^{1}H-NMR spectrum of M1-CHO in $CDCl_3$.

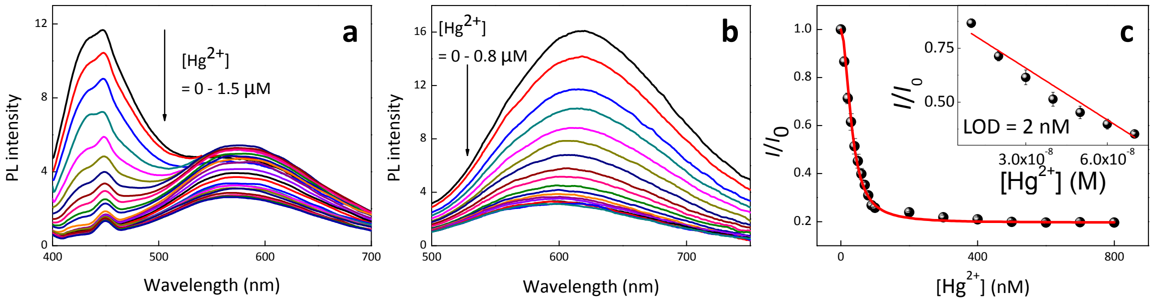

Figure S5. (**a**,**b**) PL emission spectra of M1Q in pH 4 and pH 10 with increasing [Hg^{2+}]. Excitation at 390 nm. (**c**) Intensity ratio versus [Hg^{2+}] at pH 10. Inset: linear range of titration curve varying [Hg^{2+}]. The error bars represent the standard deviation of three independent measurements. [M1Q] = 1.0×10^{-6} M in water.

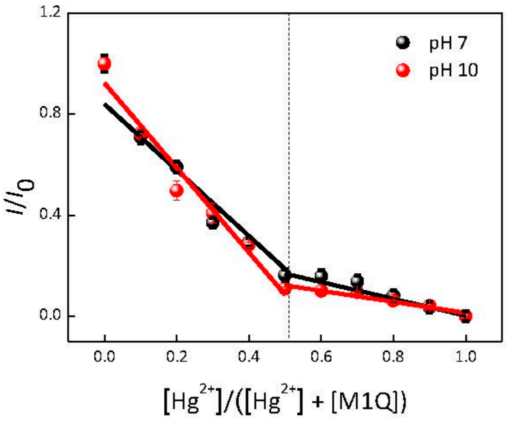

Figure S6. Job plots of M1Q in water at pH 7 and pH 10.

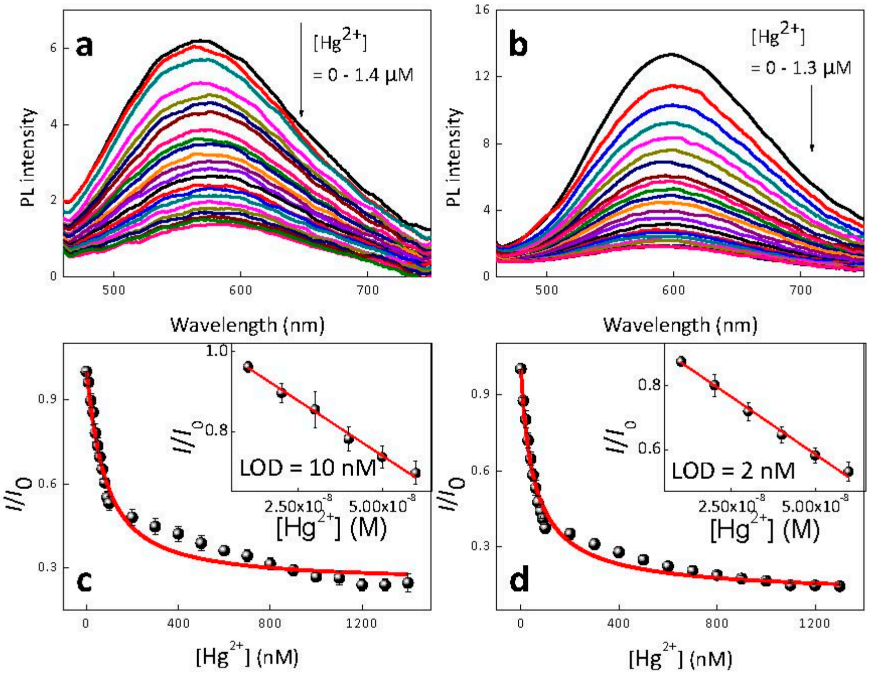

Figure S7. (**a**,**b**) PL spectra of M1Q in Han River and in tap water with increasing [Hg^{2+}]; (**c**,**d**) Normalized PL intensity (I/I_0) of M1Q with changing [Hg^{2+}]. Excitation at 390 nm. Inset: LOD determination. The error bars represent the standard deviation of three independent measurements. [M1Q] = 1.0×10^{-6} M.

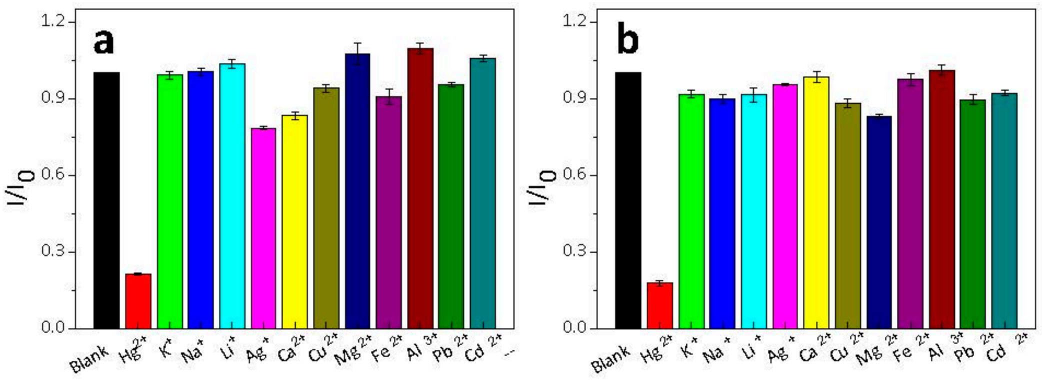

Figure S8. Selectivity test for M1Q in the Han River (**a**) and in tap water (**b**) in the presence of metal ions by excited at 390 nm. The error bars represent the standard deviation of three independent measurements. [M1Q] = 1.0×10^{-6} M; [Hg^{2+}] = 2.0×10^{-6} M; [other metal ion] = 2.0×10^{-6} M.

*** Determination of limit of detection, Hill equation and dissociation constant.**

The terms of limit of detection (LOD), Hill equation, dissociation constant, etc. are closely related to sensor characteristics. The LOD is the lowest analyte concentration that can be distinguished from the absence of that analyte [1]. The value of LOD can be calculated by the following equation: LOD = $3.3 \times \sigma$/slope. The σ is the standard deviation in the PL intensity measurements of probe itself without analytes. The slope of I/I_0 vs. [Hg^{2+}] was determined via linear fitting [2].

The Hill equation, which was originally formulated by Archibald Hill in 1910, describes the sigmoidal binding curve of analyte as a function of the analyte concentration [3]. By determining the degree of analyte binding to probe, the detection range was determined by fitting the titration curve using the Hill equation, where I/I_0 transits from 10% to 90% of its signal output [4,5].

A dissociation constant, K_d is equal to the concentration of free A when half of the total molecules of B are associated with A ($A_xB_y \rightleftharpoons$ xA + yB) [6].

The binding/dissociation equilibrium of M1/M1Q (denoted as A) and [Hg^{2+}] (denoted as B) can be described by

$$A + B \xrightarrow{k_1} AB$$

$$A + B \xleftarrow[k_{-1}]{} AB$$

k_1: association rate constant, k_{-1}: dissociation rate constant.

For the above bimolecular reaction, the equilibrium dissociation constant (K_d) can be defined as

$$K_d = \frac{k_{-1}}{k_1} = \frac{[A][B]}{[AB]}$$

or

$$\frac{[AB]}{[A]} = \frac{[B]}{K_d}$$

$$Fraction\ A\ bound = \frac{[AB]}{[A]+[AB]} = \frac{1}{\frac{[A]}{[AB]}+1} = \frac{1}{\frac{K_d}{[B]}+1} = \frac{[B]}{K_d+[B]}$$

From this expression, if Fraction A bound = [B]/(K_d + [B]) = 0.5, then [B] = K_d [7]. Thus, K_d is simply determined by measuring the Hg^{2+} concentration when the I/I_0 ratio decreases to half of the original value without Hg^{2+} (Fraction A bound = 0.5 from a titration curve).

References

1. MacDougall, D.; Crummett, W.B. Guidelines for data acquisition and data quality evaluation in environmental chemistry. *Anal. Chem.* **1980**, *52*, 2242–2249.
2. Ahuja, S.; Dong, M. (Eds.) *Handbook of Pharmaceutical Analysis by HPLC*, 6th ed.; Elsevier: London, UK, 2005.
3. Hill, A.V. The Combinations of Haemoglobin with Oxygen and with Carbon Monoxide. *J. Physiol.* **1910**, *40*, iv–vii.
4. Porchetta, A.; Vallée-Bélisle, A.; Plaxco, K.W.; Ricci, F. Using Distal-Site Mutations and Allosteric Inhibition to Tune, Extend, and Narrow the Useful Dynamic Range of Aptamer-Based Sensors. *J. Am. Chem. Soc.* **2012**, *134*, 20601–20604.
5. Jeong, J.-E.; Kim, B.; Woo, S.; Hwang, S.; Bazan, G.C.; Woo, H.Y. Principal factors that determine the extension of detection range in molecular beacon aptamer/conjugated polyelectrolyte bioassays. *Chem. Sci.* **2015**, *6*, 1887–1894.
6. Le, V.S.; Jeong, J.-E.; Kim, B.; Lee, J.; Kyhm, K.; Woo, H.Y. Inhibitor effects on molecular beacon-based mercury assays for tuning of detection range. *Sens. Actuator B Chem.* **2017**, *240*, 810–817.
7. Bisswange, H. *Enzyme Kinetics: Principles and Methods*; Wiley: Weinheim, Germany, 2008.
